# Development and Validation of an LC-MS/MS Assay for the Quantitation of MO-OH-Nap Tropolone in Mouse Plasma: Application to In Vitro and In Vivo Pharmacokinetic Studies

**DOI:** 10.3390/molecules29184424

**Published:** 2024-09-18

**Authors:** Wafaa N. Aldhafiri, Yashpal S. Chhonker, Nusrat Ahmed, Sandeep K. Singh, Staci L. Haney, James B. Ford, Sarah A. Holstein, Daryl J. Murry

**Affiliations:** 1 Department of Pharmacy Practice and Science, College of Pharmacy, University of Nebraska Medical Center, Omaha, NE 68198, USA; wafaa.aldhafiri@unmc.edu (W.N.A.); yashpal111@gmail.com (Y.S.C.); nusrat.ahmed@unmc.edu (N.A.); sasingh@unmc.edu (S.K.S.); 2 Department of Internal Medicine, University of Nebraska Medical Center, Omaha, NE 68198, USA; staci.haney@unmc.edu (S.L.H.); sarah.holstein@unmc.edu (S.A.H.); 3 Department of Pediatrics, University of Utah, Salt Lake City, UT 84113, USA; james.ford@hsc.utah.edu

**Keywords:** tropolone, in vitro metabolism, LC-MS/MS, plasma protein binding, biodistribution

## Abstract

A rapid, selective, and sensitive liquid chromatography coupled with tandem mass spectrometry (LC-MS/MS) method was developed and validated for the quantitation of MO-OH-Nap tropolone (MO-OH-Nap) in mouse plasma. MO-OH-Nap is an α-substituted tropolone with anti-proliferative properties in various cancer cell lines. Detection and separation of analytes was achieved on an ACE Excel C18 (1.7 µm, 100 × 2.1 mm, MAC-MOD Analytical, Chadds Ford, PA, USA) column with mobile phase consisting of 0.05% trifluoroacetic acid in water (mobile phase A) and 0.05% trifluoroacetic acid in acetonitrile (mobile phase B), with an isocratic elution of 15:85% (A:B) at a total flow rate of 0.25 mL/min. The LC-MS/MS system was operated at unit resolution in multiple reaction monitoring (MRM) mode, using precursor ion > product ion combination of 249.10 > 202.15 *m*/*z* for MO-OH-Nap and 305.10 > 215.05 *m*/*z* for the internal standard (IS), BA-SM-OM. The MS/MS response was linear over a concentration range of 1 to 500 ng/mL with a correlation coefficient (r^2^) of ≥0.987. The within- and between-batch precision (%RSD) and accuracy (%Bias) were within acceptable limits. The validated method was successfully applied to determine MO-OH-Nap metabolic stability, plasma protein binding, and bio-distribution studies of MO-OH-Nap in CD-1 mice.

## 1. Introduction

Tropolonoids are naturally occurring secondary metabolites found in the heartwood of Cupressaceae plants. Tropolonoids are characterized by a unique alpha-hydroxy seven-membered non-benzenoid aromatic ring, known as the tropolone nucleus [1,2,3]. Tropolones have a low molecular weight and simple structural backbone that facilitates the development of numerous α- and β-substituted structural modifications. Many tropolone derivatives have demonstrated anti-proliferative effects across various cancer cell lines, including melanoma [4,5], lymphocytic leukemia [2,6], and lung and prostate malignancies [7,8,9]. β-Thujaplicin, a well-studied tropolone derivative, has proven effective in animal tumor models, including breast cancer [10], melanoma [11], and lung cancer [12]. These key characteristics, along with acceptable toxicity in normal cells, make them potential scaffolds for drug development [1,13,14].

**Figure 1:**
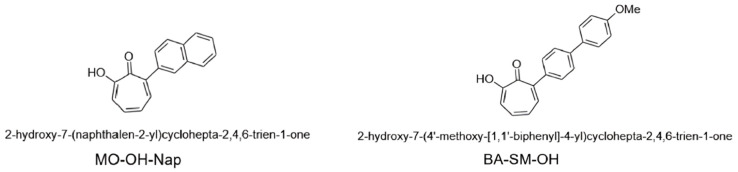
Chemical structures of the α-substituted derivatives, MO-OH-Nap and BA-SM-OH tropolone (internal standard; IS).

MO-OH-Nap tropolone (MO-OH-Nap) is a novel α-substituted tropolone (Figure 1) [15] that has cytotoxic activity against T-cell lymphocyte and myeloma cell lines following drug exposure for 48 h [15]. Additional activity has been exhibited in colon cancer cell lines, pancreatic cancer cell lines [14], and osteosarcoma cell lines following 72 h drug exposures [16].

Although the exact mechanism of action for MO-OH-Nap is not fully elucidated, the hydroxyketone in the tropolone nucleus has been shown to bind metal ions [17]. MO-OH-Nap indices cytotoxic effects in a concentration- and time-dependent manner and increased levels of apoptosis markers such as caspase 3, 8, and 9 in a manner distinct from suberoylanilide hydroxamic acid (SAHA), a pan-HDAC inhibitor [15,18]. Additionally, Ingenuity Pathway Analysis (IPA) identified MO-OH-Nap to significantly induce endoplasmic reticulum (ER) stress and activate all three arms of the unfolded protein response (UPR) in myeloma cell lines [15]. Notably, the timing of caspase cleavage induced by MO-OH-Nap differs from that triggered by SAHA, suggesting a unique mechanism of action distinct from HDAC inhibitors and may also involve its metal-chelating properties, which could influence the activity of metal-dependent enzymes within the ER [15,16,18].

Additionally, MO-OH-Nap has been found to inhibit osteosarcoma cell migration and invasion in vitro at sub-cytotoxic concentrations following 24 h incubations [16]. Metabolomic analysis identified MO-OH-Nap treatment to downregulate purine and pyrimidine nucleotide metabolic pathways following MO-OH-Nap treatment, aligning with the effects of conventional chemotherapeutics like methotrexate [16]. Additionally, in vivo dose-finding studies have demonstrated that MO-OH-Nap is well-tolerated in CD-1 mice, supporting dosages up to 9.4 mg/kg administered intravenously three times weekly, without adverse impacts on blood counts, renal and hepatic functions, or body weight [18].

These attributes indicate that MO-OH-Nap could be a promising candidate for further development in cancer treatment. However, despite its observed therapeutic promise, there remains a notable gap in the published literature regarding the in-depth bioanalytical methodologies, pre-clinical pharmacokinetics, and biodistribution properties of MO-OH-Nap and other α-substituted tropolones. Therefore, the primary aim of this research is to facilitate clinical translation by developing and validating a rapid, selective, and sensitive LC-MS/MS method for quantitating MO-OH-Nap in mouse plasma and determining the pharmacokinetics following intraperitoneal (IP) administration to mice. Furthermore, the metabolic stability, plasma protein binding (PPB), and gastrointestinal fluid stability of MO-OH-Nap were also investigated.

## 2. Results

### 2.1. Mass Spectrometric and Chromatographic Optimization

The mass spectrometric (MS/MS) conditions were optimized to improve the detection of MO-OH-Nap and IS response. The analytes responses, expressed as areas and S/N ratios, were monitored in both available ionization mode, electrospray ionization (ESI) and atmospheric pressure chemical ionization (APCI). The *m*/*z* of precursor ion (Q1) for both MO-OH-Nap and IS were scanned in both positive (+ESI) and negative (−ESI) modes. The lowest limit of detection for MO-OH-Nap and IS was achieved through the utilization of +ESI mode, which produced the best stable signal intensity compared to the −ESI mode and APCI source. The optimization of ionization energy resulted in different fragments of the protonated molecules [M + H]^+^. The highest intensity was achieved with MO-OH-Nap precursor ion (parent ion) > product ion (fragmented ion) of 249.10→202.15 *m*/*z* and 305.10→215.05 for BA-SM-OH as internal standard (IS), presented in Table 1, while MS/MS scan for both analytes was presented in Figure S1.

**Figure 2:**
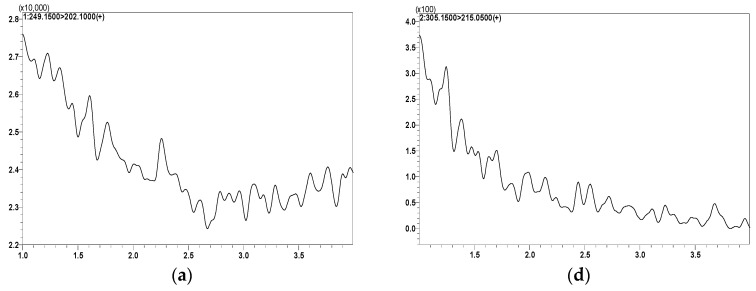

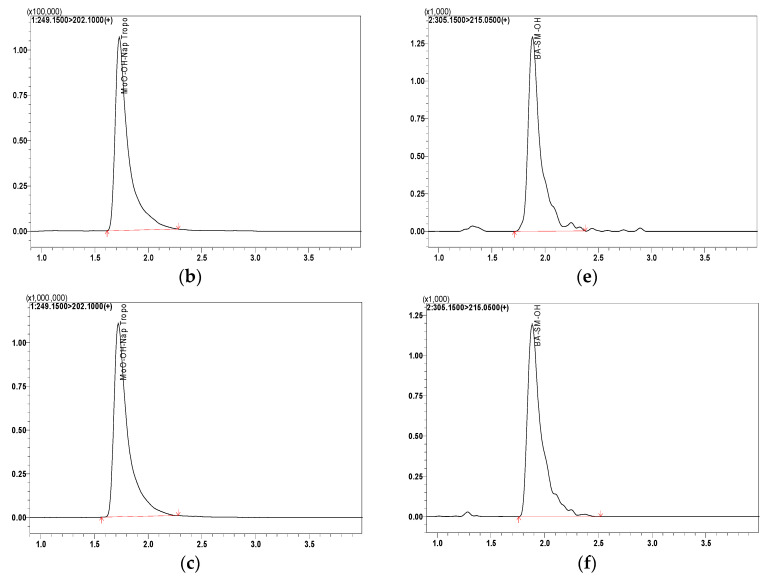
Representative MRM ion-chromatograms of MO-OH-Nap and BA-SH-OH (IS) following SPE extraction of (**a**) blank mouse plasma using the same conditions for MO-OH-Nap detection; (**b**) MO-OH-Nap spiked in mouse plasma at 10 ng/mL (retention time 1.7 min); (**c**) MO-OH-Nap mouse plasma study sample (retention time 1.7 min, after 2 h); (**d**) blank mouse plasma using the conditions for BA-SH-OH detection; (**e**) BA-SH-OH spiked in mouse plasma (retention time 1.85 min, 1.0 ng/mL); and (**f**) BA-SH-OH spiked in mouse plasma study sample after 2 h.

After optimizing MS/MS conditions, LC conditions were optimized. The various chromatographic conditions were investigated for proper elution of analytes and to attain desirable retention times, and acceptable peak shape with maximal peak resolution without interfering substances. Multiple chromatographic conditions were tested, which included several mobile phases (acetonitrile (ACN), methanol (MeOH), and water), different additives (acetic acid, trifluoroacetic acid, ammonium acetate, formic acid, and ammonium format), multiple gradient parameters, and analytical reverse phase columns (C8, C18, and C18 PFP). The optimal peak resolution and signal intensity were attained by the following separation and column conditions: ACE Excel C18 (1.7 µm, 100 × 2.1 mm) column protected with a C18 guard column. The optimal mobile phase composition was 0.05% trifluoroacetic acid (TFA) in water (mobile phase A; 15%), and 0.05% TFA in ACN (mobile phase B; 85%) operated in isocratic mode with a total flow rate of 0.25 mL/min. The short retention times were attained as MO-OH-Nap was eluted at 1.70 min and BA-SM-OH eluted at 1.85 min. The total run time was 4.0 min. The selection of BA-SM-OH as IS was appropriate to the developed method because it did not prolong the analysis time, had a similar ionization response to that of the MO-OH-Nap, and most importantly had similar chromatographic behavior. Lastly, no additional peaks were observed more than 5% at either retention time, indicating no co-elution of endogenous compounds. The representative MRM ion chromatograms are presented in Figure 2.

### 2.2. Optimization of Biosamples Extraction

Sample preparation was optimized by evaluating different extraction techniques including protein precipitation (PPT), liquid-liquid extraction (LLE), and solid phase extraction (SPE). Thus, two extraction methods consisting of PPT and SPE were optimized based on study type and matrix complexity. Both validated PPT and SPE extraction methods were reproducible and yielded consistent recovery at all the evaluated concentrations in the matrix analyzed. The PPT method was utilized for the extraction of MO-OH-Nap samples obtained from in vitro studies. SPE was utilized for the analysis of MO-OH-Nap from plasma and tissue samples due to its ability to remove phospholipids, which caused ion suppression in tissue matrices.

### 2.3. Method Validation

#### 2.3.1. Method Specificity and Sensitivity

The specificity of the developed method was assessed by analyzing a blank extracted plasma for potential interference at the MO-OH-Nap retention time (1.70 min) and BA-SM-OH (IS) retention time (1.85 min) utilizing both extraction methods (PPT and SPE). Endogenous compounds did not cause any significant interference at either retention time. Moreover, there were no co-eluted peaks that were >20% of the MO-OH-Nap lower limit of quantification (LLOQ) area, nor co-eluting peaks > 5% of the IS area in blank plasma processed with the SPE method (Figure 2), while the specificity and selectivity of the assay applying the PPT method were represented in Figure S2. The method sensitivity and selectivity were acceptable at LLOQ of 1.0 ng/mL for MO-OH-Nap.

#### 2.3.2. Standard Curve and Linearity

**Figure 3:**
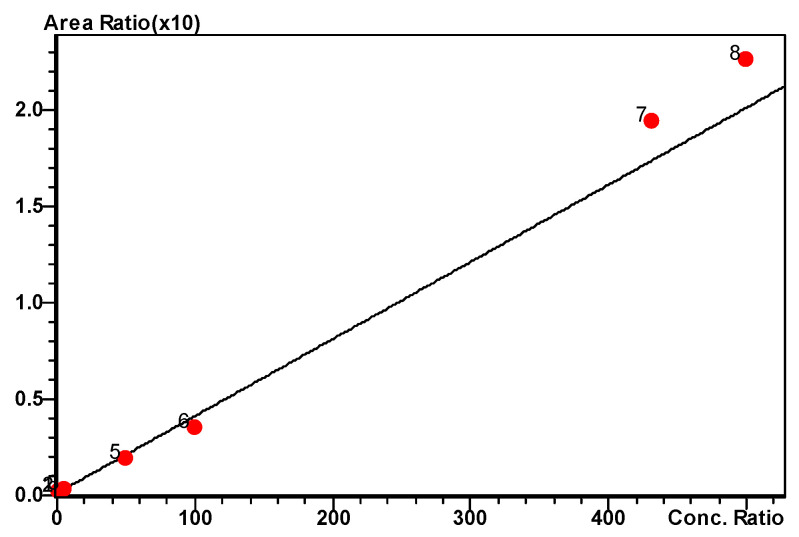
Linear relation between drug concentration and response for MO-OH-Nap Tropolone following SPE over the concentration range of 1 to 500 ng/mL, r^2^ = 0.987.

The results were fitted using a 1/x^2^ weighted linear regression of MO-OH-Nap CSs and QCs. MO-OH-Nap response was linear over the calibration curve range of 1.0 to 500 ng/mL (Figure 3). The correlation coefficient of determination (r^2^) was 0.987.

#### 2.3.3. Carry-Over

Carry-over was assessed by analyzing a blank sample (running mobile phase only) after running the 375 ng/mL—high-quality control (HQC) SPE or PPT extracted plasma. No significant peaks were observed that were ≥20% LLOQ. Thus, the carry-over effect on the method accuracy was negligible (Figures S3 and S4).

#### 2.3.4. Accuracy and Precision

Intra- and inter-day accuracy and precision were estimated for the validated method at four concentration levels in mouse plasma. 1 ng/mL—the lower limit of quantification (LLOQ), 3 ng/mL—low-quality control (LQC), 200 ng/mL—middle-quality control (MQC), and 375 ng/mL—HQC. The precision was defined as the percent relative standard deviation (%RSD). %RSD values ranged from 5.39% to 14.76% for the PPT extraction technique and from 1.50% to 17.30% for the SPE technique. The accuracy of the quantitative analysis of the compounds was defined as the percent bias (%Bias). %Bias values varied from −6.11 to 11.23 for PPT-extracted samples and −11.20 to 12.50 for SPE-extracted samples. Both precision (%RSD) and accuracy (%Bias) aligned with the acceptable ±15% of nominal concentrations, ±20% at LLOQ, for plasma and tissues that underwent PPT and SPE set by bioanalytical method validation guidelines [19]. Accuracy and precision values for MO-OH-Nap in plasma are listed in Table 2 and in tissue are listed in Table S1.

#### 2.3.5. Recovery and Matrix Effect

Mouse plasma extraction recovery of both analytes, MO-OH-Nap and IS, was calculated using three control samples (LQC—3.0 ng/mL, MQC—200 ng/mL, and HQC—375 ng/mL). The % extraction mean recovery for MO-OH-Nap following the PPT extraction method in LQC, MQC, and HQC was 79.5 ± 3.0, 92.7 ± 11.9, and 89.7 ± 7.7%, respectively, whereas the mean recovery of IS was > 90%. MO-OH-Nap extraction, following SPE, mean recovery was 80.6 ± 8.3, 54.9 ± 0.8, and 53.9 ± 6.02%, respectively at LQC, MQC, and HQC, whereas mean recovery for IS > 55%. The matrix effect (ME) was between 80 and 109%, indicating a negligible ME in extracted mouse plasma samples. The extraction recovery and ME results are tabulated in Table 3. Additionally, no significant MEs were observed in tissue samples that followed SPE (Table S2).

#### 2.3.6. Stability

MO-OH-Nap stability (lack of degradation) was assessed in the conditions that simulate storage and use conditions. These conditions are bench-top storage (up to 6 h at room temperature 21 °C), three freeze–thaw cycles (−80 °C to room temperature for 30 min, back to—80 °C, stored for 24 h, and repeated twice more), 24 h auto-sampler stability of extracted samples (at 4 °C), and long-term storage (12 months at −80 °C). MO-OH-Nap showed no considerable instability or degradation in the tested conditions. Stability sample concentrations results were all within the accepted ±15% of nominal concentrations as shown in Table 4.

#### 2.3.7. Dilution Integrity

The mean calculated concentration for samples diluted by a 2, 5, and 10 folds were within ±15% SD of the nominal concentration.

### 2.4. In Vitro Studies

#### 2.4.1. Solubility of MO-OH-Nap

The aqueous solubility of MO-OH-Nap was tested in PBS (pH 7.4), which simulated the physiological pH. MO-OH-Nap is considered partially soluble and very slightly soluble per the United States Pharmacopeia (USP) with aqueous solubility of 20.21 µg/mL (80.1 µM) [20,21].

#### 2.4.2. Blood to Plasma Ratio (B/P) of MO-OH-Nap

The (B/P) ratio was determined at a concentration of 1 µM after 0, 30, and 60 min incubations at 37 °C. (Table 5). The (B/P) ratio was 1.1, 0.9, and 1.0 after 0, 30, and 60 min incubations at 37 °C, respectively (Table 5). A (B/P) ratio ≤ 1 indicates that the drug is not binding to blood components and remains in the plasma. It also impacts the interpretation of drug concentration measurements in plasma to accurately reflect the total drug exposure in the body.

#### 2.4.3. Gastrointestinal Fluids Stability Studies of MO-OH-Nap

**Figure 4:**
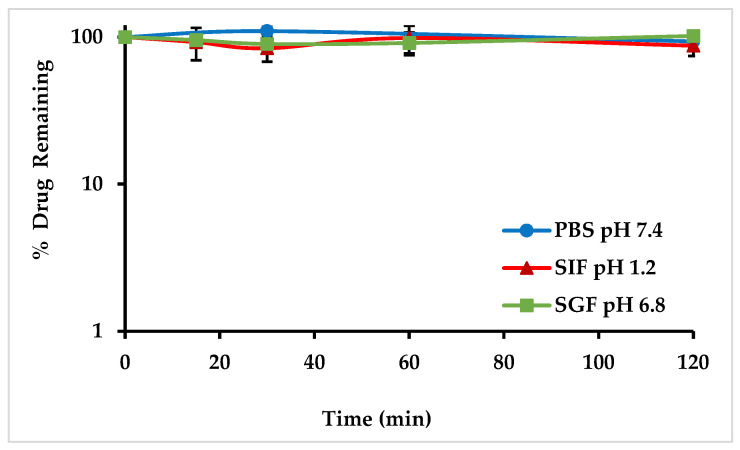
Gastrointestinal Stability of MO-OH-Nap Tropolone following a 2 h incubation in buffer PBS pH 7.4 (●), SGF pH 1.2 (▲), and SIF pH 6.8 (■).

The stability of the analyte was evaluated at gastrointestinal pH 1.2 and 6.8, as well as at physiological pH 7.4 using PBS. MO-OH-Nap was found to be stable in all gastrointestinal-simulated fluids across a wide pH range, SGF (pH 1.2), SIF (pH 6.8), and PBS (pH 7.4), with >80% of the drug remaining after 120 min incubation (Figure 4).

#### 2.4.4. Plasma Protein Binding (PPB) Study

The PPB of MO-OH-Nap was performed at two different concentrations to evaluate its concentration-dependent protein binding. MO-OH-Nap was highly bound (>99%) to mouse plasma proteins at both 1 µg/mL and 10 µg/mL concentrations (Table 6). MO-OH-Nap was stable in the Rapid Equilibrium Dialysis (RED) device under experimental conditions following a 5 h incubation. There was no observed non-specific binding of MO-OH-Nap to the dialysis chamber membrane as presented in Table 6.

#### 2.4.5. In Vitro Metabolic Stability in Mouse, Rat, and Human Liver Microsomes

**Figure 5:**
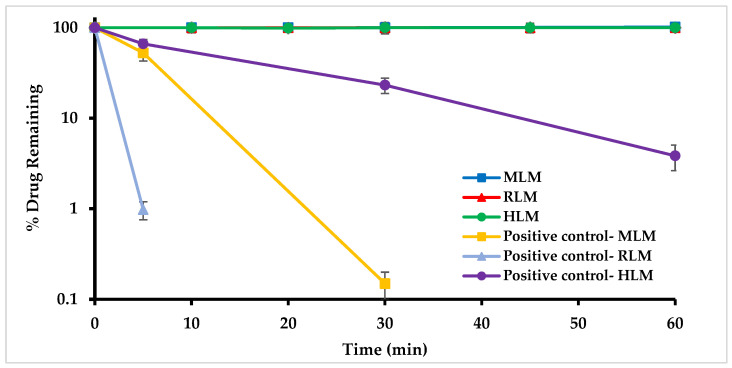
MO-OH-Nap tropolone in vitro metabolic stability in mouse (MLM, ■), rat (RLM, ▲) and human (HLM, ●) liver microsomes. MLM, RLM, and HLM lines overlap (all values are >95%). Data shown as mean ± SD (*n* = 3).

In vitro metabolic stability of MO-OH-Nap was investigated in pooled liver microsomes of mouse (MLM), rat (RLM), and human (HLM). Liver microsome stability was determined through the percentage of the parent drug remaining at different time points, relative to the parent drug at 0 min (100% parent). MO-OH-Nap showed minimal (<15%) metabolic depletion after 60 min incubation in the interspecies microsomes, as presented in Figure 5, indicating that MO-OH-Nap does not undergo CYP- or non-CYP-mediated Phase I metabolism.

### 2.5. In Vivo Pharmacokinetic Studies

#### 2.5.1. Application of Analytical Method to Pharmacokinetic Animal Study

**Figure 6:**
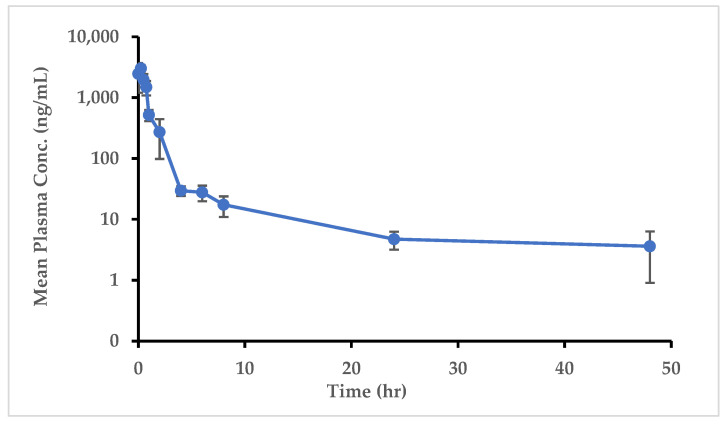
Mean plasma concentration vs. time profile of MO-OH-Nap after a single IP dose of 5 mg/kg (Mean ± S.D, *n* = 5 at each time point).

The developed and validated LC-MS/MS method was successfully applied in the quantification of MO-OH-Nap in plasma and tissue samples following a single IP dose of MO-OH-Nap of 5 mg/kg to CD1 mice. The dose was selected based on previously reported pharmacological responses in CD1 mice [18]. Plasma concentration was plotted against time as shown in Figure 6. MO-OH-Nap was detectable at 48 h in the plasma following a single dose.

The mean non-compartmental MO-OH-Nap pharmacokinetic parameters are shown in Table 7. The mean maximum observed plasma concentration (C_max_) was found to be 3040.9 ng/mL with time of maximum concentration (T_max_), averaging 0.3 h. Time zero to the last measurable concentration (AUC_0_last_) systemic exposure was 2747.9 ± 403.1 (ng×h/mL) with systemic volume of distribution (Vd/F) of 43,727.1 ± 10,870.1 (mL/kg). The observed clearance CL/F and elimination half-life (t_1/2_) of MO-OH-Nap was 1790.6 ± 305.8 mL/h/kg and 17.7 ± 7.2 h, respectively, after IP dosing.

#### 2.5.2. Tissue Distribution Study

**Figure 7:**
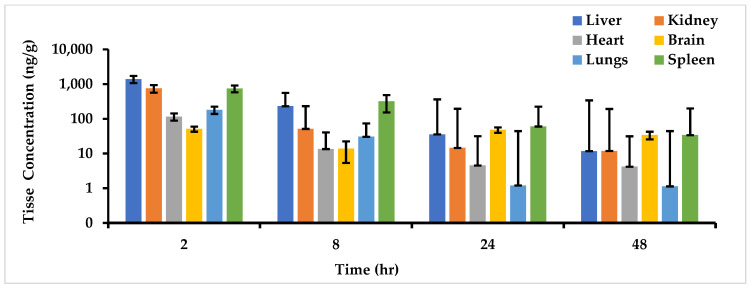
MO-OH-Nap tissue accumulation (ng/g) in liver, lungs, heart, kidney, brain, and spleen following a single 5 mg/kg IP dose to CD1 mice (Mean ± S.D, *n* = 5 at each time point).

The MO-OH-Nap SPE extraction method effectively extracted the analytes from all harvested tissues. MO-OH-Nap was detected 48 h after a single 5 mg/kg IP dose in all harvested tissue as presented in Figure 7. The highest concentration of MO-OH-Nap was observed in the liver (1391 ng/g), followed by the kidney (750 ng/g), spleen (745 ng/g), lungs (181 ng/g), heart (115 ng/g) then brain (51 ng/g) at 2 h.

## 3. Discussion

A sensitive and specific LC-ESI-MS/MS method was developed and validated for rapid and selective quantitation of MO-OH-Nap in plasma and other biomatrices. The validated bioanalytical method provided a lower limit of quantitation of 1 ng/mL (LLOQ) utilizing 100 µL of plasma, which is essential in early-phase pharmacokinetic studies. The response was linear over a concentration range of 1–500 ng/mL with a correlation coefficient (r^2^) of 0.987 or better for all calibration curves (Figure 3). This broad range supports the evaluation of the drug’s behavior at both low and high doses. This is instrumental in conducting dose-ranging studies and in establishing the therapeutic window of MO-OH-Nap. Accuracy and precision were within standard acceptance limits. The standard deviation of all back-calculated concentrations from the nominal values met the precision and accuracy acceptance criteria ±15% and ±20% for the LLOQ (Table 2 and Table S1) [19].

MO-OH-Nap and IS showed the best signal intensity, lower background noise, and acceptable sensitivity in the optimized ESI+ mode. The ESI+ generated fragment ions *m*/*z*, 249.10→202.15 for MO-OH-Nap and 305.10→215.05 *m*/*z* for BA-SM-OH showed the best reproducibility and single intensity compared to other potential ions (Table 1). This choice of ion fragment ensured that the detection is both sensitive and specific to the compounds of interest, which is crucial for accurate quantification in complex biological matrices.

Liquid chromatographic separation was achieved using a reverse phase (C18) column, which offered more affinity towards nonpolar compounds and allowed the excellent separation of the analytes of interest compared to other evaluated columns [22]. The mobile phase consisted of 0.05% TFA in water (mobile phase A; 15%), and 0.05% TFA in ACN (mobile phase A; 85%) and was operated in isocratic mode at a total flow rate of 0.25 mL/min. The use of the TFA in polar mobile systems resulted in effective chromatographic resolution with acceptable separation, high peak efficiency, and symmetry in a timely manner with a total run time of 4 min (Figure 2) [19,23,24,25]. The use of materials such as the C18 column and TFA, which are highly accessible, is a significant advantage. As it ensures that the method can be readily replicated and applied in diverse scientific settings [22].

Two extraction methods were developed and validated to optimize analyte recovery based on the type of samples being analyzed. The PPT method was effectively used for in vitro samples, such as those assessing metabolic stability, PPB, and gastrointestinal stability. The PPT extraction method was reproducible with consistent high extraction recovery averaged of >79% of MO-OH-Nap and >90% of IS with negligible ME. However, ion suppression in in vivo tissue samples required the development of an SPE extraction method for better “clean up “and reduction in ME. The ME can significantly influence the precision and accuracy of a bioanalytical method, potentially compromising the integrity of the results [26]. The SPE recovery was >53% of MO-OH-Nap and >55% of IS. Even though the SPE extraction recovery was not as high as the PPT method, it has the advantage of eliminating phospholipids present in tissue samples, therefore reducing the ME significantly and enhancing detection accuracy (Table 3). The MO-OH-Nap response was linear over the calibration curve range of 1.0 to 500 ng/mL in mouse plasma and all tissue matrices. The method was fully validated for mouse plasma and liver tissue that underwent SPE while partial validation was followed for other tissue utilizing SPE. The accuracy response of QCs in biological matrices (plasma and all tissue homogenates) met acceptance criteria, which confirms its reliability for accurate quantification of MO-OH Nap in in vivo samples [27].

The stability of MO-OH-Nap was tested under typical laboratory storage and use conditions to ensure the compound’s reliability throughout experimental and clinical processes [19,28]. The MO-OH-Nap was stable under all the tested use and handling conditions ensuring reproducible results and easy handling (Table 4). MO-OH-Nap was stable in all gastrointestinal simulated fluids across a wide pH range, SGF (pH 1.2), SIF (pH 6.8), and PBS (pH 7.4), indicating the potential for oral route administration with >80% parent remaining after 120 min incubation (Figure 4). The ability to maintain stability in varied gastrointestinal conditions suggests that MO-OH-Nap can withstand the acidic environment of the stomach and the variable pH levels throughout the digestive tract, which is essential for effective oral drug delivery.

The presented method was successfully implemented in the processing of (B/P) ratio and PPB in vitro assay, which is a key pharmacokinetic parameter that is used in understanding a drug tissue distribution, and the prediction of the free drug concentration at the site of action. MO-OH-Nap (B/P) ratio was ≤1 over an hour, which indicates that the drug is not binding to blood components and remains in the plasma [29]. It also impacts the interpretation of drug concentration measurements in plasma accurately reflecting the total drug exposure in the body (Table 5). MO-OH-Nap was highly bound (>99%) to mouse plasma proteins in a concentration-independent manner (Table 6). MO-OH-Nap is considered partially soluble and very slightly soluble per the United States Pharmacopeia (USP) with an aqueous solubility of 20.21 µg/mL (80.1 µM) [20,21]. These findings align with dose-escalation studies where the solubility limit of the drug was identified at a dose of 9.4 mg/kg. Multi-dose testing (9.4 mg/kg intravenously three times weekly) in CD-1 mice has previously demonstrated the lack of adverse effects on blood counts, renal and hepatic functions, or body weight [18]. However, poor solubility and high protein bound do not preclude the use of MO-OH-Nap due to its promising cytotoxic and highly potent activity [14,18].

MO-OH-Nap stability in mouse S9 fraction was previously reported [18]. MO-OH-Nap showed similar stability when evaluated in MLM, RLM, and HLM (Figure 5) suggesting no phase I and phase II metabolism. Therefore, MO-OH-Nap elimination is presumed to be extrahepatic and non-CYP mediated.

The developed method was successfully applied in the quantification of MO-OH-Nap in plasma and tissue samples, over 48 h, after a single 5 mg/kg IP administration. The Cmax was 3041 ng/mL, at a very short time averaging 0.3 h Tmax, suggesting that the drug MO-OH-Nap is rapidly absorbed into systemic circulation following IP administration. The t_1/2_ of 17.7 h indicates that MO-OH-Nap stays in the system for a substantial duration and could support less frequent dosing. The AUC_0_last_ and infinity extrapolated AUC_0_∞_ show a small difference between them, implying that the drug is mostly cleared by the last measured time point. The Vd/F of MO-OH-Nap was 43,277.1 mL/kg as compared to the total body water of mice, i.e., 580 mL/kg, suggesting its extensive distribution into the tissues from the vascular compartment. The MO-OH-Nap CL/F,1790.6 mL/h/kg, is less than the mean liver blood flow (4320 mL/h/kg) indicating its moderately low extraction, which is consistent with its long half-life [30,31,32]. MO-OH-Nap was detected in all tested tissues with high perfusion to the liver and the spleen (Figure 7). Overall, these findings show that MO-OH-Nap is absorbed rapidly with moderate to slow elimination and widely distributed in different tissues.

## 4. Materials and Methods

### 4.1. Mass Spectrometric and Chromatographic Conditions Optimization

MO-OH-Nap (purity: ≥99%) and BA-SM-OH (purity: ≥99%), used as an IS, were generously provided by Dr. Dennis Wright (Department of Pharmaceutical Sciences, University of Connecticut, Storrs, CT, USA). TFA, LCMS grade MeOH, and ACN were purchased from ThermoFisher Scientific (Fair Lawn, NJ, USA). Ultrapure water was obtained using a Barnstead GenPure water purification system (ThermoFisher Scientific; Fair Lawn, NJ, USA). Strata-X-AW 33 µm Polymeric Weak Anion cartridges (30 mg per 1 mL) were purchased from Phenomenex Inc. (Torrance, CA, USA). An ACE Excel C18 column (1.7 µm, 100 × 2.1 mm) was purchased from MAC-MOD Analytical (Chadds Ford, PA, USA). The column was protected with a C18 guard column purchased from Phenomenex (Torrance, CA, USA). Mouse plasma was purchased from Equitech-Bio, Inc. (Kerrville, TX, USA). All additional materials and reagents used in this study were of analytical grade or higher as they were purchased from standard chemical suppliers.

### 4.2. Liquid Chromatographic and Mass Spectrometric (LC-MS/MS) Conditions for MO-OH-Nap

Assay method development was conducted using an LC-MS/MS 8060 system (Shimadzu Scientific Instruments, Columbia, MD, USA) equipped with a dual ion source (DUIS) interface operated in positive electrospray ionization mode (ESI+). LabSolutions LCMS software Version 5.9 (Shimadzu Scientific, Inc., Columbia, MD, USA) was used for data acquisition and quantitation. The compound-dependent MS parameters, such as temperature, voltage, gas pressure, etc., were optimized by auto method optimization for MO-OH-Nap, BA-SM-OH (IS), and α-phenyl tropolone (secondary IS) using a 1.0 µg/mL stock solution of each individual compound in MeOH. Finale instrument conditions and detection parameters are summarized in Table 8.

The MS/MS system was operated at unit resolution in the multiple reaction monitoring (MRM) mode, using precursor ion→product ion combinations of 249.10→202.15 *m*/*z* for MO-OH-Nap and 305.10→215.05 *m*/*z* for BA-SM-OH as depicted in Table 1.

### 4.3. Preparation of Stock, Calibration Standards and Quality Control Samples

The primary stock solutions at 1 mg/mL of MO-OH-Nap and IS were prepared in the mixture of DMSO and MeOH (1:1, *v*:*v*), respectively. The working stock solutions were subsequently diluted with MeOH to the concentration of the calibration standards (CSs) solution: 0.01, 0.02, 0.05, 0.1, 0.5, 1, 4.32, 5 µg/mL. All CSs were stored at −20 °C until use. Mouse plasma was used to prepare calibration curve samples by spiking 50 µL of mouse plasma with 5 µL of the appropriate stock to achieve the following CSs: 1, 2, 5, 10, 50, 100, 432, and 500 ng/mL. The same manner was applied to prepare the four different quality control (QC) samples: 1 ng/mL—LLOQ, 3 ng/mL—LQC, 200 ng/mL—MQC, and 375 ng/mL—HQC. All CSs and QCs were prepared freshly when needed. On the other hand, a working stock solution of IS was prepared at a fixed concentration of 1 µg/mL in MeOH to ensure consistent IS concentration throughout the assay. All the standard stock solutions were stored at −20 °C until use.

### 4.4. Sample Preparation and Extraction Method

Two extraction methods were developed and validated for the extraction of analytes depending on the type of samples. PPT extraction technique was used for the extraction of in vitro samples such as metabolic stability samples, PPB samples, and gastrointestinal stability samples. SPE extraction technique was used for extraction of analytes from plasma and tissue samples that requires better “clean up” and reduction in ME. Additionally, CSs and QCs samples were also processed by both methods and validated.

In vitro samples were extracted utilizing the PPT technique by spiking 5 µL of appropriate calibration stock in 50 µL blank bio-matrix followed by the addition of 10 µL of the IS stock (1.0 µg/mL) in a 1.5 mL-centrifuge tube. The samples were vortexed for 30 s before adding 300 µL ice-cold ACN to initiate protein precipitation. Then, the mixture was vortexed for 2 min, followed by centrifugation for 15 min at 14,000 rpm (22,000× *g*) at 4 °C. Thereafter, 100 µL of the supernatant was transferred to an HPLC vial. An aliquot of 10 µL was injected into the LC–MS/MS system for analysis.

In vivo PK samples were processed using the SPE technique. In brief, blank bio-matrix (100 µL) was spiked with 10 µL of appropriate working stock and 10 µL of IS stock (1.0 µg/mL). After spiking, samples were vortexed for 30 s at 1000 rpm. Afterward, 600 µL of ACN was added followed by 1 min vortex at 1000 rpm. Then 600 µL of 4% phosphoric acid (pH 1.65 ± 0.1) was added to the above solution and vortexed for 2 min, then centrifuged at 1400× *g* for 10 min. Samples were then centrifuged for 10 min at 3500 rpm (1400× *g*) and the supernatant was separated and collected to load into the conditioned SPE Cartridge, Strata-X-AW 33 µm polymeric weak anion cartridge (Phenomenex Inc., Torrance, CA, USA). The SPE cartridge was conditioned with 1 mL of ACN followed by 1 mL of 2% formic acid (pH 2.08 ± 0.1). Samples were loaded after conditioning and washed with 1 mL 15% ACN. Analytes were eluted two times with 1 mL 10% ammonium hydroxide (NH4OH) in ACN (pH 12.25 ± 0.1), then dried in TurboVap^®^ concentrator (Thermo Scientific, Asheville, NC, USA) and reconstituted with 100 µL of the mobile phase of 0.05% TFA in ACN: 0.05% TFA in water (B 85: A 15). Thereafter, the supernatant was transferred to an HPLC vial and 10 µL was injected into LC–MS/MS system for analysis.

### 4.5. Method Validation

To ensure the robustness of the developed bioanalytical LC-MS/MS assay, the FDA 2022 guidelines for bioanalytical method validation were followed in the development and validation of the method selectivity, sensitivity, LLOQ, accuracy, precision, and ME [19].

The selectivity and specificity of the validated method were assessed by comparing the chromatograms of six different blank mouse plasma samples with that MO-OH-Nap and IS spiked plasma. To determine the method sensitivity the signal-to-noise ratio (S/N) approach was used to define the methods LLQC. A 3:1 S/N for the lower limit of detection (LLOD) and a 10:1 S/N for the LLOQ is considered sufficient to discriminate the analyte from the background noise.

The standards calibration curves were constructed by linearly plotting the relationship between the response: peak area ratio (MO-OH-Nap/IS) on the *y*-axis versus the concentration of the analyte (MO-OH-Nap) on the *x*-axis using a 1/x^2^ weighing factor. The calibration curve consists of: A blank sample (plasma sample processed without IS), a zero sample (plasma sample containing IS only), and a 12 non-zero concentrations consisting of eight CSs and four QCs. The four QC levels are: LLOQ (1 ng/mL), LQC (3 ng/mL), MQC (200 ng/mL) and HQC (375 ng/mL). The response of each CS and QC was held to be identifiable, discrete, and reproducible with a precision and accuracy of ±15% standard deviation (SD) of their expected value, except for the LLOQ being held to ±20% of its expected value. CSs were run in ascending order followed by two consecutive “zero samples” to assess the impact of any carry-over. Carry-over must not exceed 20% of the expected S/N of LLOQ.

Freshly prepared QCs were prepared in the determination of the method intra-day and inter-day accuracy and precision. By analyzing variations in the QCs at four different concentrations (LLQC, LQC, MQC, and HQC) in mouse plasma, the deviation of the mean measurement from the nominal value serves as the measure of accuracy and precision. The analysis was performed in six replicates over three consecutive/independent days. Precision (%RSD) with acceptance criteria of ±15% for all QC except for LLOQ being allotted a ±20 %RSD. %RSD was calculated as described in Equation (1). Accuracy (%Bias) with the same acceptance criteria as precision. %Bias was calculated as described in Equation (2).

(1) $$\%RSD=\frac{\left(standard\;deviation\right)}{mean}\times 100$$

(2) $$\%Bias=\frac{\left(observed\;conc.-nominal\;conc.\right)}{nominal\;conc}\times 100$$

#### 4.5.1. Recovery and Matrix Effect

Extraction recovery was calculated for MO-OH-Nap at three different QC concentrations (LQC 3 ng/mL), (MQC 200 ng/mL) and (HQC 375 ng/mL) and IS at a concentration of 1 µg/mL by comparing the mean peak area of the analyte spiked before extraction to the mean peak area of the same analyte spiked post-extraction.

The ME was evaluated at three QC levels. The blank mouse plasma was processed, as described in Section 2.4, and then the post-extract matrix was spiked with the analyte-prepared equivalent to the QCs. The mean peak area of the analytes spiked in the blank matrix was compared with neat QCs samples that were prepared in MeOH. The absolute ME was calculated as described in Equations (3) and (4).

(3) $$ME=\frac{Mean\;Peak\;area\;of\;analyte\;spiked\;post-extraction}{Mean\;Peak\;area\;of\;analyte\;in\;Solvent}\times 100$$

(4) $$IS\;ME=\frac{Mean\;Peak\;area\;ratio\;of\;IS\;spiked\;post-extraction}{Mean\;Peak\;area\;ratio\;of\;IS\;in\;Solvent}\times 100$$

#### 4.5.2. Stability

The chemical stability of MO-OH-Nap and IS in plasma samples were assessed at LQC, MQC, and HQC concentrations, *n* = 3, under the following storage conditions: bench-top storage (up to 6 h at room temperature, 21 ± 0.5 °C), three freeze–thaw cycles (−80 ± 0.5 °C to room temperature for 30 min, back to −80 ± 0.5 °C then stored for 24 h, the cycle was repeated twice more), long-term storage (12 months at −80 ± 0.5 °C), and 24 h auto-sampler stability of extracted samples (at 4 ± 0.5 °C).

#### 4.5.3. Dilution Integrity

The dilution integrity of samples was tested on six replicates at three different levels of dilution: two- (2 × HQC), five- (5 × HQC), and ten-fold (10 × HQC) dilutions of the highest QC concentration. The calculated concentration measurements were compared to the nominal concentration at each dilution level. The precision and accuracy of the dilution integrity sample must fall within ±15% SD of the nominal concentration.

### 4.6. In Vitro Studies

#### 4.6.1. Aqueous Solubility of MO-OH-Nap

Thermodynamic aqueous solubility of the MO-OH-Nap was assessed in phosphate buffer saline (PBS, 100 mM pH 7.4) by dissolving 1 mg MO-OH-Nap in PBS (1 mL) and stirred in a shaking incubator for 24 h at ambient temperature (37 °C). After completion of incubation, the microcentrifuge tube was centrifuged at 10,000× *g* for 10 min. The supernatant (100 μL) was mixed with an equal volume of ACN and centrifuged again at 10,000× *g* for 10 min. The supernatant was further diluted 80× times with mobile phase and transferred to autosampler vials and analyzed by LC-MS/MS.

#### 4.6.2. Blood to Plasma Ratio (B/P) of MO-OH-Nap

Fresh mouse blood (800 µL) was incubated in a water bath maintained at 37 °C for 10 min prior to drug spiking. Blood was spiked with the drug at concentrations of 1 µM to maintain organic content < 1%. Aliquots of 50 µL were collected at different time points (0, 30, and 60 min) for blood analysis as well an additional sample of 120 µL was collected into a micro-centrifuge tube and centrifuged at 4000× *g* for 10 min at 4 °C to separate 50 µL plasma for analysis. For matrix match and calibration, blank plasma of 50 µL was added to the collected blood aliquot, and 50 µL of blank blood was added to the extract of 50 µL plasma. The whole blood and plasma samples were further processed by PPT as described above. The analyte peak area ratios were used to calculate the (B/P) ratio using Equation (5).

(5) $$(B/P)\;ratio=\frac{Blood\;Concentration\;}{Plasma\;Concentration}$$

#### 4.6.3. Gastrointestinal Fluid Stability Studies

The gastric stability of MO-OH-Nap was determined at different physiological pH conditions using simulated gastric/intestinal fluid (SGF/SIF) or PBS (pH7.4) prepared with USP specifications [33]. Briefly, simulated gastric fluid (SGF), pH 1.2, was prepared by dissolving 0.2 g of sodium chloride (NaCl) and 3.2 g of pepsin in 7 mL hydrochloric acid (HCl) and a sufficient amount of water to make 1000 mL. The simulated intestinal fluid (SIF), pH 6.8, was prepared by dissolving 6.805 g of monobasic potassium phosphate in 250 mL of water. Then 77 mL of 0.2 N sodium hydroxide (NaOH) was mixed with 500 mL water and 10 g of pancreatin, then made up to 1000 mL. The SIF solution is adjusted to pH 6.8 ± 0.1 with either 0.2 N NaOH or 0.2 N HCl. MO-OH-Nap at 1 µg/mL concentration was incubated with SGF, SIF and PBS (100 mM pH 7.4) at 37 °C on a shaking water bath, *n* = 3. Samples (100 µL) were collected at 0, 15, 30, 60, and 120 min. Immediately after sample collection, 300 µL ACN was added as a quenching solvent to stop chemical degradation. IS solution (10 µL from 1 µg/mL stock) was added to each sample, vortexed the centrifuged at 14,000 rpm (22,000× *g*) and 4 °C, and then transferred to HPLC vial for LC-MS/MS analysis. Stability was determined through the percentage of the parent drug remaining at different time points relative to the parent drug at 0 min (100% parent).

#### 4.6.4. Plasma Protein Binding (PPB) Study

The plasma protein binding (PPB) assay was conducted at 0.25 µg/mL and 2.5 µg/mL (1 and 10 μM) concentration of MO-OH-Nap using a RED device system (Thermo Scientific, Rockford, IL, USA). The RED Kit represents 90 wells with two chambers at each well (red chamber for plasma and white buffer chamber separated by Teflon base plate. Prior to the experiments, RED kit was rinsed with 10% ethanol for 10 min and left to dryness. Also, mouse plasma was centrifuged at 2200 rpm (540× *g*) for 5 min and adjusted to pH 6.7 with lactic acid. In brief, MO-OH-Nap spiked plasma samples (300 µL) were added in red chamber. The buffer chamber contained 350 mL of Phosphate-buffered saline (100 mM, pH 7.4), which contained 100 mM sodium phosphate (Na_3_PO_4_) and 150 mM NaCl. The RED kit plate was covered with sealing tape and incubated at 37 °C on an orbital shaker at 300 rpm for 5 h. Aliquot of 50 µL were collected from both chambers, then mixed with equal amount of buffer or blank plasma, consecutively, for analysis. The samples were then processed using PPT extraction technique, as per described in the Section 4.4, and analyzed by LC-MS/MS.

Additionally, the plasma stability and the non-specific binding potential of MO-OH-Nap to the RED kit dialysis membrane were determined separately at the same time from the remaining plasma samples spiked with MO-OH-Nap.

Stability and non-specific binding were assessed by collecting samples at 0 h and 5 h of spiked MO-OH-Nap buffer that was incubated in the same PPB condition for 5 h. The two samples were then analyzed by LC-MS/MS and the concentration was then compared between the two time points. The stability, equilibrium, device recovery, and PPB in plasma were calculated using the following equations:

(6) $$\%\;Drug\;Bound=\frac{{Concentration}_{donor\;cell}-{Conentration}_{receiver\;cell}}{{Concentration}_{donor\;cell}}\times 100$$

(7) $$\%\;Device\;Recovery=\frac{{Concentration}_{receiver\;cell}+{Concentration}_{donor\;cell}}{{Concentration}_{5\;h}}\times 100$$

(8) $$\%\;Remaining\;at\;5\;h=\frac{{Concentration}_{5\;h}}{{Concentration}_{0\;h}}\times 100$$

(9) $$Equilibrium\;at\;5\;h=\frac{{Concentration}_{receiver\;cell}}{{Concentration}_{donor\;cell}}$$

#### 4.6.5. In Vitro Metabolic Stability

Metabolic stability was assessed using mouse, rat, and human liver microsomes (XenoTech, LLC, Lenexa, KS, USA) for phase I metabolism. Briefly, A reaction solution was prepared by mixing 450 µL of PBS (100 mM, pH 7.4), 25 µL of microsomal protein (20 mg/mL), MgCl_2_ (5 µL; 10 mM), and 50 µL of NADPH (2 mM). The final volume (0.5 mL) was pre-incubated for 10 min in a 37 °C water bath at 60 rpm. An aliquot of MO-OH-Nap (2 µL from 1 µg/mL stock) was spiked into the above solution to initiate the reaction. Aliquots of (50 µL) were collected at time intervals (0, 5, 15, 20, 30, 45, and 60 min) and quenched with 300 µL of ACN, then spiked with 10 µL of IS stock (1.0 µg/mL). Afterwards, 10 µL of IS stock (1.0 µg/mL) was spiked in each sample then vortexed (5 min) and centrifuged at 10,800 rpm (13,000× *g*) for 15 min. Samples were vortexed and centrifuged at 10,800 rpm (13,000× *g*) for 15 min, the supernatant was collected and transferred to an HPLC vial and 10 µL were injected into the LC-MS/MS system. Testosterone and diclofenac were used as positive and negative controls to ensure that liver microsomes and incubation conditions were appropriate for conducting metabolism studies.

### 4.7. In Vivo Pharmacokinetic Studies

#### 4.7.1. Biodistribution Study, Animals, Drug Administration and Sampling

Animal studies were carried out in accordance with the Guide for the Care and Use of Laboratory Animals (U.S. National Institutes of Health) and were approved by the University of Nebraska Medical Center (UNMC) Institutional Animal Care and Use Committee (IACUC) (protocol number 17-014-04-FC).

Female CD-1 mice,6–8-week-old, with weights ranging from 20 to 25 g were housed in the UNMC animal facility, at a temperature of 23–24 °C, relative humidity of 40–70%, and 12/12-h light/dark cycles. Mice were left to acclimatize to laboratory conditions for 7 days prior to experimental day.

MO-OH-Nap was dissolved in a 5% DMSO, 45% *w*/*v* hydroxypropyl-β-cyclodextrin solution in PBS (final concentration 0.625 mg/mL MO-OH-Nap). MO-OH-Nap (5 mg/kg) was administered IP. After dosing, approximately 100 µL of blood was collected in Lithium-Heparin-Microvette 300 µL Capillary Blood Collection Tube (SARSTEDT AG& Co., Nümbrecht, Germany) from the maxillary vein at 5, 15, 30 min and 1, 2, 4, 6, 8, 24, and 48 h. Two blood time points were collected from every mouse and the third time point was the terminal time point, for a total of three time points from each mouse (5 mice/group/per time point). Blood was centrifuged at 4000× *g* at 4 °C for 10 min to extract plasma. The collected plasma samples were stored at −80 °C until analysis.

The specified tissues including liver, lungs, kidney, spleen, heart, and brain were collected at terminal times of 2, 8, 24 and 48 h after dosing. Tissue samples were rinsed with PBS to remove excess blood and blotted with filter paper. After weighing, each tissue sample was individually homogenized with de-ionized water using a TissueLyser II (Qiagen Science, Louisville, KY, USA). A dilution factor of five was used for liver, spleen, brain, lungs, and kidney while the heart was homogenized at a 6-fold dilution factor. All tissues were stored at −80 °C until analysis. Plasma concentrations (ng/mL) and tissue concentrations (ng/g) were determined for each time point collected using LC-MS/MS analysis.

#### 4.7.2. Data Analysis

The pharmacokinetics of MO-OH-Nap were determined using non-compartmental (NCA) pharmacokinetics analysis, utilizing Phoenix^®^ WinNonlin software version 8.2 (Certara Corporation, Mountain View, CA, USA). The Cmax and Tmax were determined from visual inspection of the concentration time data. The AUC_0_∞_ was estimated using the linear trapezoidal method from 0 time point to last time point (0-tlast) and extrapolation from last time point to infinity based on the observed concentration at the last time point divided by the terminal elimination rate constant (k). The t_1/2_, CL/F and Vd/F were calculated using Equation (10), Equation (11) and Equation (12), respectively.

(10) $$t_{1/2}=\frac{0.693}{k}$$

(11) $$CL=\frac{Dose}{{AUC}_{0\_\infty }}$$

(12) $$Vd=\frac{Dose\;}{k\times {AUC}_{0\_\infty }}$$

## 5. Conclusions

In this report, a robust, rapid, selective, and sensitive, LC-MS/MS method was successfully established for the quantitation of MO-OH-Nap in mouse plasma and tissues. MO-OH-Nap was found stable in gastric and intestinal fluid with high protein binding and had no CYP-mediated metabolism. Additionally, MO-OH-Nap was shown to be widely distributed with long plasma half-life and tissue accumulation following IP dosing in mice.

Future research will assess the efficacy of MO-OH-Nap in inhibiting tumor growth in mouse models of osteosarcoma. Additionally, it will be crucial to design innovative drug delivery systems that can address the solubility challenges of MO-OH-Nap and improve its PK. Overall, these findings will assist in the further development of MO-OH-Nap as a lead candidate against osteosarcoma.

## Figures and Tables

**Table 1:** Summary of MS/MS parameters: precursor ion, fragment ions, voltage potential (Q1), collision energy (CE), and voltage potential (Q3) for MO-OH-Nap and BA-SM-OH.

Analytes	MRM Transition *m*/*z* (Q1→Q3)	Q1 (V)	Q3 (V)	CE (V)	Retention Time (min)
MO-OH-Nap (Target)	249.10→202.15	−100	−23	−35	1.70
	249.10→231.10	−11	−23	−24	
BA-SM-OH (IS)	305.10→215.05	−23	−22	−25	1.85
	305.10→195.05	−13	−25	−21	

**Table 2:** Accuracy and precision. Intra- and inter-assay accuracy and precision of MO-OH-Nap tropolone in plasma (*n* = 6).

Extraction Techniques	Nominal Conc. (ng/mL)	Accuracy		Precision	
		%Bias Intra–Assay	%Bias Inter–Assay	%RSD Intra–Assay	%RSD Inter–Assay
PPT	LLOQ (1 ng/mL)	−5.03	11.23	9.45	14.76
	LQC (3 ng/mL)	−1.90	−5.09	5.39	6.10
	MQC (200 ng/mL)	−6.11	0.62	7.15	9.51
	HQC (375 ng/mL)	1.64	2.12	11.73	14.67
SPE	LLOQ (1 ng/mL)	5.40	12.50	1.80	17.30
	LQC (3 ng/mL)	−4.00	1.80	1.50	13.40
	MQC (200 ng/mL)	−6.10	−3.70	13.40	10.80
	HQC (375 ng/mL)	−9.20	−11.20	11.60	11.20

**Table 3:** Assessment of the recovery and matrix effect of MO-OH-Nap Tropolone in mouse plasma, (Mean ± S.D, *n* = 3).

Extraction Techniques	Nominal Conc. (ng/mL)	MO-OH-Nap Tropolone		BA-SM-OH (IS)	
		Mean Extraction Recovery (%)	Mean ME (%)	Mean Extraction Recovery (%)	Mean ME (%)
PPT	LQC (3 ng/mL)	79.5 ± 3.0	93.12 ± 10.86	90.8 ± 10.8	89.64 ± 21.52
	MQC (200 ng/mL)	92.7 ± 11.9	90.29 ± 20.55		
	HQC (375 ng/mL)	89.7 ± 7.7	86.02 ± 25.83		
SPE	LQC (3 ng/mL)	80.6 ± 8.3	97.28 ± 6.75	55.9 ± 9.2	90.59 ± 6.82
	MQC (200 ng/mL)	54.9 ± 0.8	94.26 ± 1.74		
	HQC (375 ng/mL)	53.8 ± 6.0	109.49 ± 10.99		

**Table 4:** Stability of MO-OH-Nap was tested in mouse plasma at different storage conditions, (Mean ± S.D, *n* = 3).

Nominal Conc. (ng/mL)	Measured Mean Conc. (ng/mL)	% Accuracy
Bench-top stability at 21 °C, up to 6 h, Mean ± S.D, *n* = 3.		
LQC (3 ng/mL)	3.2 ± 0.5	107.8 ± 14.8
MQC (200 ng/mL)	191.6 ± 18.1	95.8 ± 9.1
HQC (375 ng/mL)	380.5 ± 20.9	101.5 ± 5.6
Freeze-thaw stability at −80 °C, up to 3 Cycle, Mean ± S.D, *n* = 3.		
LQC (3 ng/mL)	3.0 ± 0.4	100.5 ± 13.3
MQC (200 ng/mL)	167.9 ± 1.8	84.0 ± 0.9
HQC (375 ng/mL)	332.2 ± 17.6	88.6 ± 4.7
Autosampler stability at 4 °C, up to 24 h, Mean ± S.D, *n* = 3.		
LQC (3 ng/mL)	3.1 ± 0.2	101.9 ± 5.8
MQC (200 ng/mL)	180.2 ± 1.10	90.1 ± 0.6
HQC (375 ng/mL)	340.2 ± 20.1	90.7 ± 5.3
Long-term stability at −80 °C, 12 months, Mean ± S.D, *n* = 3.		
LQC (3 ng/mL)	2.9 ± 0.1	95.6 ± 2.4
MQC (200 ng/mL)	199.5 ± 1.3	99.8 ± 0.6
HQC (375 ng/mL)	347.4 ± 1.5	92.7 ± 0.4

**Table 5:** MO-OH-Nap Tropolone Blood to plasma ratio (B/P) in mouse plasma, Mean ± SD, *n* = 3.

Time (min)	MO-OH-Nap B/P Ratio
0	1.1 ± 0.1
30	0.9 ± 0.2
60	1.0 ± 0.12

**Table 6:** MO-OH-Nap Tropolone Plasma protein binding in mouse plasma following a 5 h incubation, Mean ± SD, *n* = 3.

Nominal Conc. of MO-OH-Nap (µg/mL)	% Plasma Protein Bound ± SD	% Device Recovery ± SD	% Drug Remaining at 5 h ± SD	Equilibrium at 5 h ± SD
1 µg/mL	99.6 ± 0.1	103.5 ± 18.7	105.6 ± 0.8	0.3 ± 0.5
10 µg/mL	99.7 ± 0.1	119.4 ± 6.6	91.1 ± 2.5	0.1 ± 0.00

**Table 7:** Pharmacokinetic parameters of MO-OH-Nap after a single IP MO-OH-Nap dose of 5 mg/kg (Mean ± S.D, and CV%, *n* = 20). C_max_: maximum observed concentration, T_max_: time of maximum observed concentration, t_1/2_: terminal half-life, AUC_0_∞_: AUC from time of dosing extrapolated to infinity, AUC_0_last_: Area under the moment curve from the time of dosing to the last measurable concentration, Vd/F: Volume of distribution, CL/F: Total body clearance.

Pharmacokinetic Parameters	Mean	S.D	CV%
C_max_ (ng/mL)	3040.9	1088.4	35.8
T_max_ (h)	0.3	0.2	56.4
t_1/2_ (h)	17.7	7.2	40.6
AUC_0_∞_ (h × ng/mL)	2859.8	499.3	17.5
AUC_0_last_ (h × ng/mL)	2747.9	403.1	14.7
Vd/F (mL/kg)	43,727.1	10,870.1	24.9
CL/F (mL/h/kg)	1790.6	305.8	17.1

**Table 8:** Liquid chromatographic and mass spectrometric (LC-MS/MS) conditions.

Parameter	Condition
LC-MS/MS model	LC-MS/MS 8060 system (Shimadzu Scientific Instruments, Columbia, MD, USA) equipped with a dual ion source (DUIS)
LC-MS/MS Software	LabSolutions LCMS software Version 5.9 (Shimadzu Scientific, Inc., Columbia, MD, USA)
LC-MS/MS Ionization mode	ESI+ mode
MS parameters	Nebulizing Gas: 2.0 L/min Drying gas flow: 10.0 L/min Heating gas flow: 10.0 L/min Interface Temp: 300 °C DLTemp: 250 °C Block Heater Temp: 400 °C
Column	ACE Excel C18 (1.7 µm, 100 × 2.1 mm, Advanced Chromatography Technologies, Ltd., Aberdeen, UK) protected with a C18 guard column (Phenomenex, Torrance, CA, USA)
Mobile phase A	Water with 0.05% *v*/*v* TFA
Mobile phase B	ACN with 0.05% *v*/*v* TFA
Flow	0.25 mL/min
Isocratic elution	(15% A: 85% B)
Total run time	4 min
Injection volume	10 µL

## Data Availability

Data are contained within the article and Supplementary Materials.

## Supplementary Materials

The following supporting information can be downloaded at: https://www.mdpi.com/article/10.3390/molecules29184424/s1, Figure S1. MS/MS spectra of MO-OH-Nap tropolone (a) and BA-SM-OH (IS) (b); Figure S2. MRM ion-chromatograms of MO-OH and IS following PPT extraction of (a) blank mouse plasma using the same conditions for MO-OH-Nap detection, (b) MO-OH-Nap spiked in mouse plasma at 10 ng/mL (retention time 1.7 min), (c) MO-OH-Nap mouse plasma study sample (retention time 1.7 min, after 1 h), (d) blank mouse plasma using the conditions for BA-SH-OH (IS) detection. (e) BA-SH-OH spiked in mouse plasma (retention time 1.85 min, 200 ng/mL) and (f) BA-SH-OH spiked in mouse plasma study sample after 1 h; Figure S3. Chromatograms of blank sample (running just the mobile phase) using the same conditions for MO-OH-Nap detection after running the SPE HQC spiked (a) MO-OH-Nap in plasma, (b) BA-SH-OH in plasma, (c) MO-OH-Nap in liver tissue homogenate, (b) BA-SH-OH in liver tissue homogenate; Figure S4. Chromatograms of blank sample (running just the mobile phase) using the same conditions for MO-OH-Nap detection after running the PPT HQC spiked (a) MO-OH-Nap in plasma, (b) BA-SH-OH in plasma, (c) MO-OH-Nap in liver tissue homogenate, (b) BA-SH-OH in liver tissue homogenate; Table S1. Accuracy and precision. Intra–and inter–assay accuracy and precision of MO-OH-Nap tropolone in liver tissue homogenates (*n* = 6); Table S2: Mean matrix effect of MO-OH-Nap tropolone mice liver tissue homogenates following SPE extraction (*n* = 3).

## References

[1] Zhao J. (2007). Plant troponoids: Chemistry, biological activity, and biosynthesis. Curr. Med. Chem..

[2] Ononye S.N., Vanheyst M.D., Giardina C., Wright D.L., Anderson A.C. (2014). Studies on the antiproliferative effects of tropolone derivatives in Jurkat T-lymphocyte cells. Bioorganic Med. Chem..

[3] Cao F., Orth C., Donlin M.J., Adegboyega P., Meyers M.J., Murelli R.P., Elagawany M., Elgendy B., Tavis J.E. (2018). Synthesis and Evaluation of Troponoids as a New Class of Antibiotics. ACS Omega.

[4] Sugawara K., Ohbayashi M., Shimizu K., Hatori M., Kamei H., Konishi M., Oki T., Kawaguchi H. (1988). BMY-28438 (3,7-dihydroxytropolone), a new antitumor antibiotic active against B16 melanoma. I. Production, isolation, structure and biological activity. J. Antibiot..

[5] Tomita K., Hoshino Y., Nakakita Y., Umezawa S., Miyaki T., Oki T., Kawaguchi H. (1989). BMY-28438 (3,7-dihydroxytropolone), a new antitumor antibiotic active against B16 melanoma. II. Taxonomy of producing organism. J. Antibiot..

[6] Li J., Falcone E.R., Holstein S.A., Anderson A.C., Wright D.L., Wiemer A.J. (2016). Novel α-substituted tropolones promote potent and selective caspase-dependent leukemia cell apoptosis. Pharmacol. Res..

[7] Liu S., Yamauchi H. (2006). Hinokitiol, a metal chelator derived from natural plants, suppresses cell growth and disrupts androgen receptor signaling in prostate carcinoma cell lines. Biochem. Biophys. Res. Commun..

[8] Liu L., Zhu H., Wu W., Shen Y., Lin X., Wu Y., Liu L., Tang J., Zhou Y., Sun F. (2019). Neoantimycin F, a Streptomyces-Derived Natural Product Induces Mitochondria-Related Apoptotic Death in Human Non-Small Cell Lung Cancer Cells. Front. Pharmacol..

[9] Hsiao C.J., Hsiao S.H., Chen W.L., Guh J.H., Hsiao G., Chan Y.J., Lee T.H., Chung C.L. (2012). Pycnidione, a fungus-derived agent, induces cell cycle arrest and apoptosis in A549 human lung cancer cells. Chem. Biol. Interact..

[10] Chen S.M., Wang B.Y., Lee C.H., Lee H.T., Li J.J., Hong G.C., Hung Y.C., Chien P.J., Chang C.Y., Hsu L.S. (2017). Hinokitiol up-regulates miR-494-3p to suppress BMI1 expression and inhibits self-renewal of breast cancer stem/progenitor cells. Oncotarget.

[11] Huang C.-H., Lu S.-H., Chang C.-C., Thomas P.A., Jayakumar T., Sheu J.-R. (2015). Hinokitiol, a tropolone derivative, inhibits mouse melanoma (B16-F10) cell migration and in vivo tumor formation. Eur. J. Pharmacol..

[12] Huang C.H., Jayakumar T., Chang C.C., Fong T.H., Lu S.H., Thomas P.A., Choy C.S., Sheu J.R. (2015). Hinokitiol Exerts Anticancer Activity through Downregulation of MMPs 9/2 and Enhancement of Catalase and SOD Enzymes: In Vivo Augmentation of Lung Histoarchitecture. Molecules.

[13] Nozoe T. (1951). Substitution products of tropolone and allied compounds. Nature.

[14] Ononye S.N., VanHeyst M.D., Oblak E.Z., Zhou W., Ammar M., Anderson A.C., Wright D.L. (2013). Tropolones as lead-like natural products: The development of potent and selective histone deacetylase inhibitors. ACS Med. Chem. Lett..

[15] Haney S.L., Allen C., Varney M.L., Dykstra K.M., Falcone E.R., Colligan S.H., Hu Q., Aldridge A.M., Wright D.L., Wiemer A.J. (2017). Novel tropolones induce the unfolded protein response pathway and apoptosis in multiple myeloma cells. Oncotarget.

[16] Haney S.L., Feng D., Kollala S.S., Chhonker Y.S., Varney M.L., Williams J.T., Ford J.B., Murry D.J., Holstein S.A. (2024). Investigation of the activity of a novel tropolone in osteosarcoma. Drug Dev. Res..

[17] Bryant B.E., Fernelius W.C., Douglas B.E. (1953). Formation Constants of Metal Complexes of Tropolone and Its Derivatives. I. Tropolone1. J. Am. Chem. Soc..

[18] Haney S.L., Varney M.L., Safranek H.R., Chhonker Y.S., G-Dayanandan N., Talmon G., Murry D.J., Wiemer A.J., Wright D.L., Holstein S.A. (2019). Tropolone-induced effects on the unfolded protein response pathway and apoptosis in multiple myeloma cells are dependent on iron. Leuk. Res..

[19] Food and Drug Administration (2022). Bioanalytical Method Validation Guidance for Industry.

[20] Alsenz J., Kansy M. (2007). High throughput solubility measurement in drug discovery and development. Adv. Drug Deliv. Rev..

[21] Censi R., Di Martino P. (2015). Polymorph Impact on the Bioavailability and Stability of Poorly Soluble Drugs. Molecules.

[22] Moldoveanu S.C., David V., Moldoveanu S.C., David V. (2013). Chapter 5—Retention Mechanisms in Different HPLC Types. Essentials in Modern HPLC Separations.

[23] Sonawane L.V., Poul B.N., Usnale S.V., Waghmare P.V., Surwase L.H. (2014). Bioanalytical method validation and its pharmaceutical application-a review. Pharm. Anal. Acta.

[24] Simeoli R., Cairoli S., Greco M., Bellomo F., Mancini A., Rossi C., Dionisi Vici C., Emma F., Goffredo B.M. (2024). A New and Rapid LC-MS/MS Method for the Determination of Cysteamine Plasma Levels in Cystinosis Patients. Pharmaceuticals.

[25] Wang Y.-H., Mondal G., Butawan M., Bloomer R.J., Yates C.R. (2020). Development of a liquid chromatography-tandem mass spectrometry (LC–MS/MS) method for characterizing caffeine, methylliberine, and theacrine pharmacokinetics in humans. J. Chromatogr. B.

[26] Côté C., Bergeron A., Mess J.-N., Furtado M., Garofolo F. (2009). Matrix Effect Elimination During LC–MS/MS Bioanalytical Method Development. Bioanalysis.

[27] Aldhafiri W.N., Chhonker Y.S., Zhang Y., Coulter D.W., McGuire T.R., Li R., Murry D.J. (2020). Assessment of tissue distribution and metabolism of MP1, a novel pyrrolomycin, in mice using a validated LC-MS/MS method. Molecules.

[28] Thakare R., Chhonker Y.S., Gautam N., Alamoudi J.A., Alnouti Y. (2016). Quantitative analysis of endogenous compounds. J. Pharm. Biomed. Anal..

[29] Singh S.K., Valicherla G.R., Joshi P., Shahi S., Syed A.A., Gupta A.P., Hossain Z., Italiya K., Makadia V., Singh S.K. (2018). Determination of permeability, plasma protein binding, blood partitioning, pharmacokinetics and tissue distribution of Withanolide A in rats: A neuroprotective steroidal lactone. Drug Dev. Res..

[30] Valicherla G.R., Riyazuddin M., Shahi S., Gupta A.P., Syed A.A., Husain A., Gayen J.R. (2020). LC-ESI-MS/MS assay development and validation of a novel antidiabetic peptide PSTi8 in mice plasma using SPE: An application to pharmacokinetics. J. Pharm. Biomed. Anal..

[31] Davies B., Morris T. (1993). Physiological parameters in laboratory animals and humans. Pharm. Res..

[32] Singh H., Satish N., Babu T.R., Singh A., Yadav B., Singh S.K., Wahajuddin M., Siddiqui M.I., Jagavelu K., Sudhakar G. (2024). Functionalized Azirine Based Scaffolds as Endothelin Inhibitors for the Selective Anti-Angiogenic activity. Eur. J. Med. Chem..

[33] Klein S. (2010). The use of biorelevant dissolution media to forecast the in vivo performance of a drug. Aaps J..

