# Supplementary material for: Development and Validation of an LC-MS/MS Assay for the Quantitation of MO-OH-Nap Tropolone in Mouse Plasma: Application to In Vitro and In Vivo Pharmacokinetic Studies
Source: Molecules. 2024 Sep 18;29(18):4424. doi: 10.3390/molecules29184424 (PMC11434026; doi:10.3390/molecules29184424)
Supplement: Supplementary file 1 [file molecules-29-04424-s001.zip › molecules-3139031-supplementary.pdf]

Supplementary File

Development and validation of a LC-MS/MS assay for the quantitation of MO-OH-Nap Tropolone in mouse plasma: Application to in-vitro and in-vivo pharmacokinetic studies

Wafaa N. Aldhafiri[1], Yashpal S. Chhonker [1,†], Nusrat Ahmed[1], Sandeep K. Singh[1], Staci L. Haney[2], James B Ford[3], Sarah A. Holstein[2], Daryl J Murry[1,*]

[1]Department of Pharmacy Practice and Science, College of Pharmacy, University of Nebraska Medical Center, Omaha, NE 68198, USA. [2]Department of Internal Medicine, University of Nebraska Medical Center, Omaha, NE 68198, USA. [3]Department of Pediatrics, University of Utah, Salt Lake City, UT 84113, USA.
[†]Current address: Frontage Laboratories, Exton, PA 19341, USA.

*Correspondence: dj.murry@unmc.edu; Tel.: 4025593790

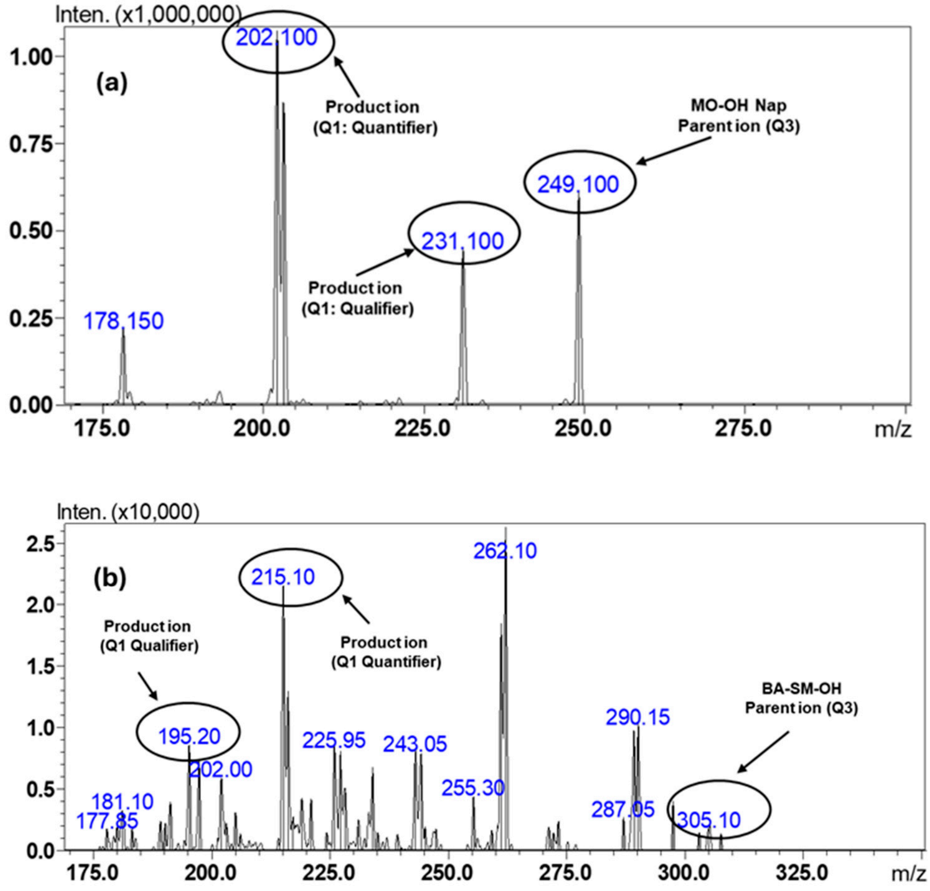

Figure S1. MS/MS spectra of MO-OH-Nap tropolone (a) and BA-SM-OH (IS) (b).

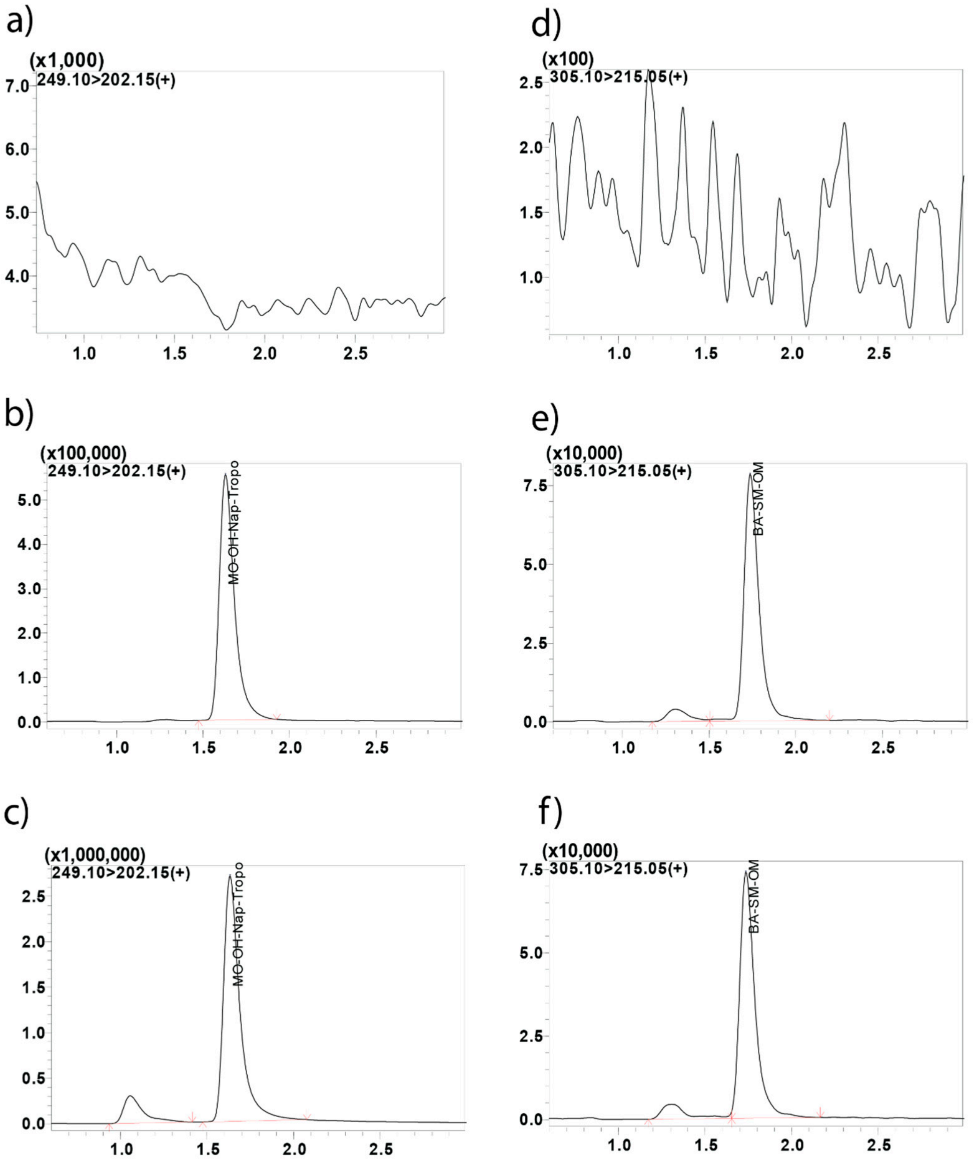

Figure S2. MRM ion-chromatograms of MO-OH and IS following PPT extraction of (a) blank mouse plasma using the same conditions for MO-OH-Nap detection, (b) MO-OH-Nap spiked in mouse plasma at 10 ng/mL (retention time 1.7 min), (c) MO-OH-Nap mouse plasma study sample (retention time 1.7 min.), (d) blank mouse plasma using the conditions for BA-SH-OH (IS) detection.(e) BA-SH-OH spiked at 200 ng/mL in mouse plasma (retention time 1.85 min,) and (f) BA-SH-OH spiked at 200 ng/mL in mouse plasma study sample (retention time 1.85 min).

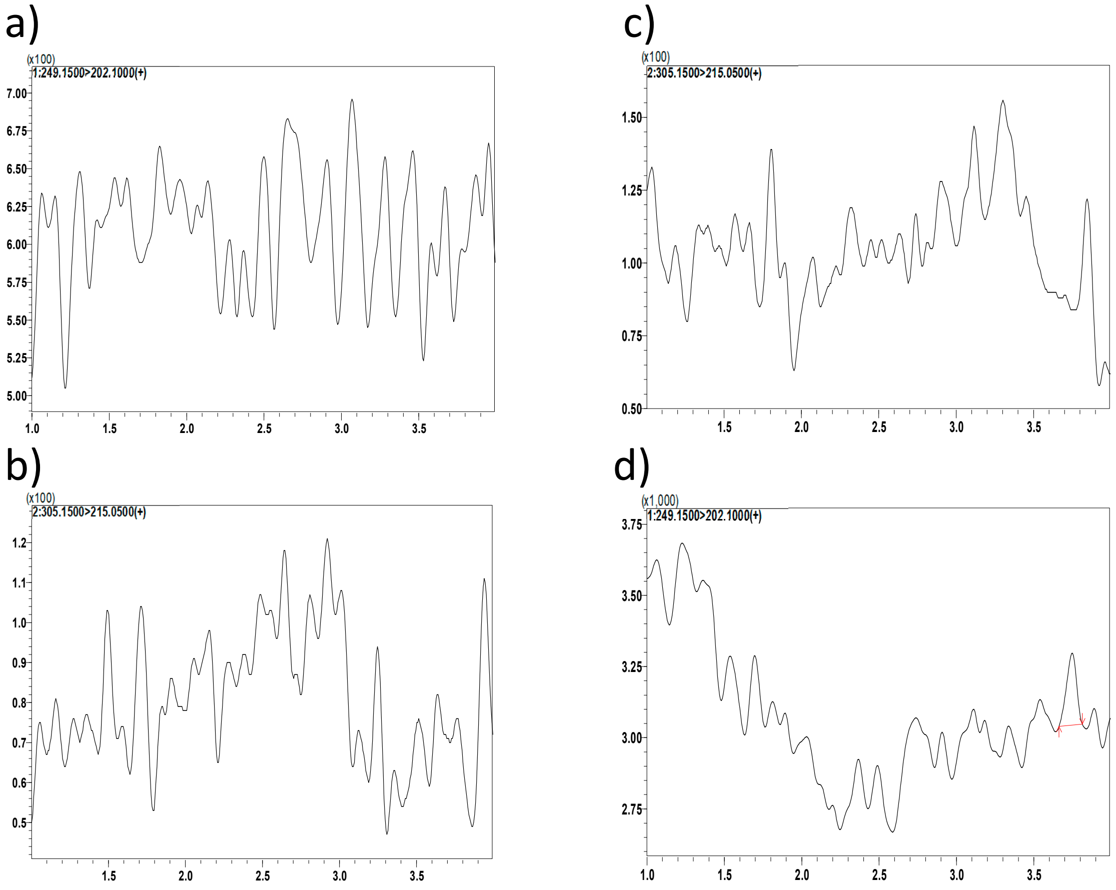

Figure S3. Chromatograms of blank sample using the same conditions for MO-OH-Nap detection after running the SPE HQC spiked (a) MO-OH-Nap in plasma, (b) BA-SH-OH in plasma, (c) MO-OH-Nap in liver tissue homogenate, (b) BA-SH-OH in liver tissue homogenate.

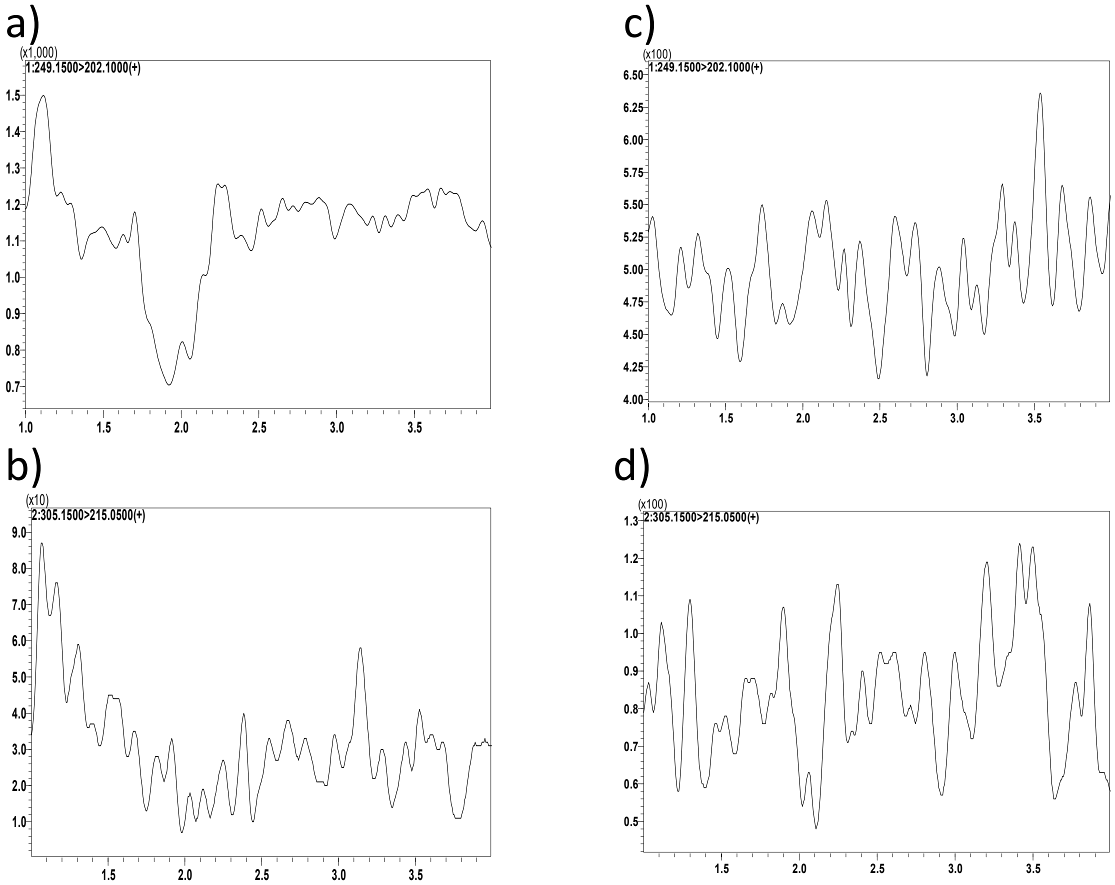

Figure S4. Chromatograms of blank sample using the same conditions for MO-OH-Nap detection after running the PPT HQC spiked (a) MO-OH-Nap in plasma, (b) BA-SH-OH in plasma , (c) MO-OH-Nap in liver tissue homogenate, (b) BA-SH-OH in liver tissue homogenate.

Table S1. Accuracy and precision. Intra–and inter–assay accuracy and precision of MO-OH-Nap tropolone in liver tissue homogenates (n = 6).

Extraction techniques	Nominal Conc. (ng/mL)	Accuracy		Precision	
		%Bias intra–assay	%Bias inter–assay	%RSD intra–assay	%RSD inter–assay
SPE	LLOQ (1.0 ng/mL)	9.34	12.20	1.61	1.12
	LQC (3.0 ng/mL)	1.83	2.79	0.49	4.52
	MQC (100 ng/mL)	13.03	-14.77	13.78	9.19
	HQC (375 ng/mL)	11.36	-4.06	8.00	13.48

Table S2: Mean matrix effect of MO-OH-Nap tropolone mice liver tissue homogenates following SPE extraction (n=3).

Extraction techniques	Nominal Conc. (ng/mL)	MO-OH-Nap Tropolone	BA-SM-OH (IS)
		Mean Matrix effect (%)	Mean Matrix effect (%)
SPE	LQC (3.0 ng/mL)	102.45 ±2.24	99.22 ±2.22
	MQC (100 ng/mL)	111.06 ± 17.71	
	HQC (375 ng/mL)	84.00 ±12.43	
